# Case Report: Long-Term Response to Radiotherapy Combined With Targeted Therapy in Histiocytic Sarcoma Harboring Mutations in MAPK and PI3K/AKT Pathways

**DOI:** 10.3389/fonc.2021.755893

**Published:** 2021-12-06

**Authors:** Zijian Liu, Yin Xiao, Xinxiu Liu, Qiuhui Li, Tao Liu, Fang Zhu, Gang Wu, Liling Zhang

**Affiliations:** Cancer Center, Union Hospital, Tongji Medical College, Huazhong University of Science and Technology, Wuhan, China

**Keywords:** histiocytic sarcoma, radiotherapy, MAPK pathway, PI3K/Akt pathway, imatinib

## Abstract

**Background:**

Histiocytic sarcoma (HS) is a rare hematopoietic malignancy with an aggressive clinical presentation associated with a poor overall survival. To date, surgical resection, radiation therapy, and chemotherapy were often utilized for HS, but curative effects are rather disappointing.

**Case Presentation:**

A 19-year-old female was referred to our hospital with a pathologic diagnosis of HS in December 2017. The patient had a severe airway obstruction resulting from a large mass (6.0 cm × 4.4 cm) arising from the left parapharyngeal space. She did not respond to cyclophosphamide, doxorubicin, vincristine, prednisone, and etoposide (CHOEP) chemotherapy, then she was switched to radiotherapy and crizotinib according to next-generation sequencing (NGS) results (mutations in MET and MAP2K1). The patient got a partial response after radiotherapy and crizotinib, then she switched to imatinib combined with thalidomide treatment. The patient got a long-term complete response from the treatment and is alive 44 months after initial diagnosis without disease progression. Further KEGG pathway enrichment analysis of NGS results from patient’s tissue revealed that phosphatidylinositol 3′ kinase (PI3K)/AKT and mitogen-activated protein kinase (MAPK) pathways were activated in this HS patient. We further performed experiments *in vitro* in a canine histiocytic sarcoma cell line DH82, in order to explore the possible mechanism of imatinib plus thalidomide in HS. Results of cell counting kit-8 (CCK8) assays showed that the proliferation activity of DH82 was significantly inhibited by imatinib but not thalidomide. Combined thalidomide and imatinib treatment did not improve the inhibitory effects of imatinib to DH82. Results of Western blot confirmed the inhibitory effects of imatinib on DH82 by targeting activation of MAPK and PI3K/AKT pathways.

**Conclusion:**

Radiotherapy combined with targeted therapy guided by NGS may be promising, and further perspective clinical trial is warranted for the localized HS.

## Introduction

Histiocytic sarcoma (HS), a subset of histiocytic and dendritic cell neoplasms, is a rare hematopoietic malignancy with an aggressive clinical presentation associated with a median overall survival of 6 months (1, 2). To date, given the limited consensus on the treatment of this type of low occurrence disease, existing data showed that non-Hodgkin lymphoma treatment approaches including surgical resection and radiation therapy (3) for local control, chemotherapy for systemic therapy were often utilized for HS, but results are rather disappointing (4). Here, we describe a patient who had HS with mutations in mitogen-activated protein kinase (MAPK), and the phosphatidylinositol 3′ kinase (PI3K)/AKT pathways obtained a long-term response to radiotherapy and targeted therapy.

## Case Presentation

**Figure 1:**
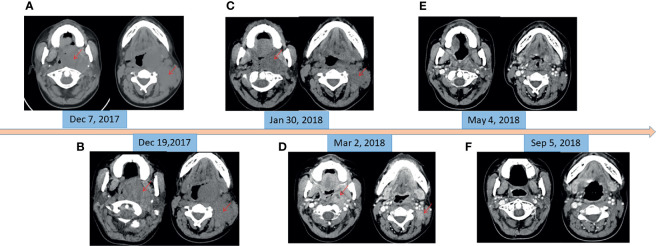
CT imaging of changes of masses in oropharynx and left neck. **(A)** Before chemotherapy (pharyngeal mass: 6.0 cm × 4.6 cm; neck mass: 3.9 cm × 2.9 cm). **(B)** At the beginning of radiotherapy and 11 days after chemotherapy (pharyngeal mass: 6.0 cm × 4.4 cm; neck mass: 3.5 cm × 2.8 cm). **(C)** At the end of the radiotherapy and oral crizotinib for 1 month (pharyngeal mass: 4.3 cm × 2.7 cm; neck mass: 2.6 cm × 1.4 cm). **(D)** At the beginning of oral imatinib and thalidomide (pharyngeal mass: 2.8 cm × 1.9 cm; neck mass: 2.1 cm × 1.4 cm). **(E)** Oral imatinib and thalidomide for 2 months. **(F)** Oral imatinib and thalidomide for 6 months.

**Figure 2:**
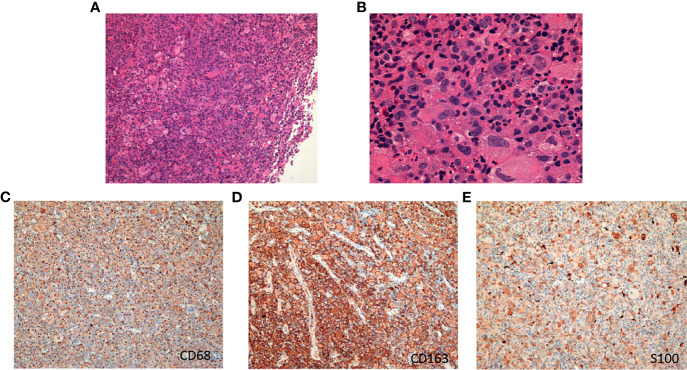
The hematoxylin-eosin (H&E) and immunohistochemical pictures of the tumor. **(A)** H&E, original magnification, ×100. **(B)** H&E, original magnification, ×400. **(C)** Immunohistochemical staining for CD68. **(D)** Immunohistochemical staining for CD163. **(E)** Immunohistochemical staining for S100.

In December 2017, a 19-year-old female was referred to our hospital with a recent diagnosis of histiocytic sarcoma. The patient had presented as pharyngeal pain and left neck mass with fever for 2 months. She had received tracheotomy because of airway obstruction resulting from the large mass arising from the left parapharyngeal space at local clinic. PET/CT scan showed a large solid mass located in the left parapharyngeal space with compression of pharyngeal cavity and multiple enlarged lymph nodes in the left neck (**Figure 1A**). The histopathological review confirmed diagnosis of HS. Histologically, HS is composed of large polygonal cells with epithelioid-to-pleomorphic morphology, abundant eosinophilic to vacuolated or foamy cytoplasm, ovoid to irregularly shaped nuclei, and variably prominent nucleoli (**Figures 2A, B**). For immunohistochemical markers, most HS express CD68 and CD163 and partially express S100 (**Figures 2C–E**). Chemotherapy (cyclophosphamide, doxorubicin, vincristine, prednisone, and etoposide (CHOEP)) was initially started on December 8, 2017 with the aim of stabilization of the fulminate disease course; however, the lesions did not shrink and pain and dysphagia were heavier caused by the compression of the pharynx (**Figure 1B**). In order to relieve the compression, irradiation to the lesions of pharyngeal and neck was started on day 11 of chemotherapy. After irradiation of 20 Gy/10 F, the size of lesions became a little bit smaller (pharyngeal mass: from 6.0 cm × 4.4 cm to 5.6 cm × 4.0 cm; neck mass: from 3.5 cm × 2.8 cm to 2.9 cm × 2.6 cm).

Meantime, next-generation sequencing (NGS) of tumor tissue was performed using a panel of 93 genes (Gene^+^ OncoLym). This analysis revealed the presence of oncogenic mutation c.2888-1G>T in the *MET* gene, exon 14 (allele frequency, 5.58%), as well as an activated mutation c.361T>A (C121S) in the *MAP2K1* gene, exon 3 (allele frequency, 17.23%). MEK inhibitor trametinib has been reported to be effective in HS patients with MAP2K1 mutation (5); however, trametinib was not available in China at that time. It has been reported that patients with MET exon 14 skipping mutation-positive nonsmall cell lung cancer are sensitive to MET inhibitor crizotinib (6); therefore, this histiocytic sarcoma patient began to take crizotinib (250 mg, twice daily) after irradiation of 20 Gy/10 F. Partial response (PR) was observed after radiotherapy of a total dose of 60 Gy/30 F and 1-month treatment of crizotinib, with sum of the product of the longest perpendicular dimensions (SPD) decreased by 57% (**Figure 1C**). The lesions kept shrinking (**Figure 1D**) after radiotherapy, and crizotinib was still taken daily for 1 month more. However, 2-month treatment of crizotinib cost her family RMB 100,000 Yuan. The patient could no longer afford such an expensive drug.

In order to search for new targetable therapeutic drugs, NGS was done again with a panel of 1,021 genes (Gene^+^ Onco-C1021T). The most frequently mutated genes were mutation c.410G>A (G137D) in the *DUSP2* gene, exon 2 (allele frequency, 18.9%), mutation c.290G>A (C97Y) in the *HIST1H3B* gene, exon 1 (allele frequency, 15.9%), and mutation c.3646A>T (S1216C) in the *GRIN2A* gene, exon 13 (allele frequency, 15.7%). Both DUSP2 and GRIN2A are in the RET signaling pathway. Imatinib is a tyrosine kinase inhibitor (TKI) that inhibits RET, PDGFR, and KIT. It has been reported to be effective in some HS cases (7). The patient was subsequently treated with imatinib (400 mg daily) and thalidomide (100 mg daily) since March 2018. The cost of imatinib and thalidomide was RMB 2,600 Yuan/month. Two months after the treatment, excellent PR was observed (**Figure 1E**) compared with tumor size in March 2018. Four months later the re-evaluation by CT scans showed a nearly complete remission (CR) (**Figure 1F**). The patient took maintenance of imatinib and thalidomide for 2 years and stopped the treatment in March 2020. To date (September 2021), 45 months after HS diagnosis, she is still alive without tumor recurrence.

**Figure 3:**
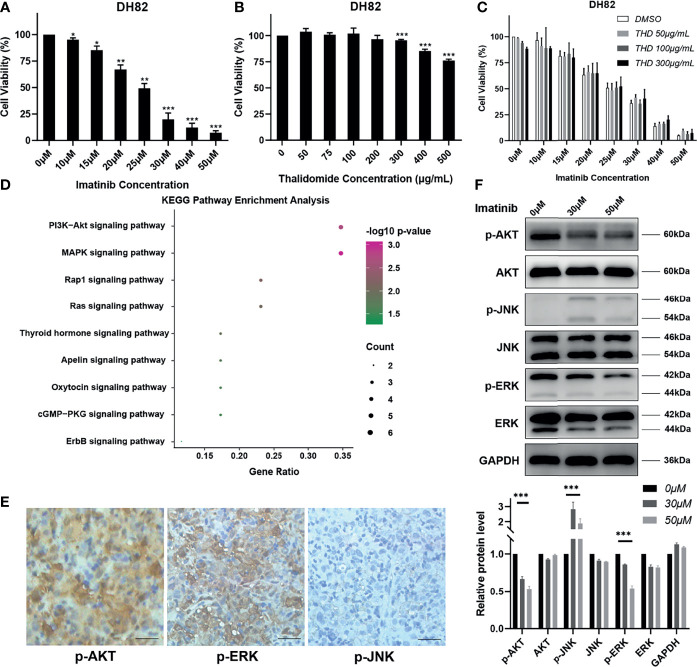
Imatinib could inhibit MAPK and PI3K/AKT pathways *in vitro*. **(A–C)** Cells were exposed to different concentrations of imatinib, thalidomide (THD), and imatinib + thalidomide. Cell viability was assessed using CCK8 assays after treatment for 24 h. Data were obtained from three independent experiments. ^*^ *p* < 0.05, ^**^ *p* < 0.01, ^***^ *p* < 0.001 vs. each control group. **(D)** KEGG pathway enrichment analysis of gene mutations in patient. **(E)** Representative immunohistochemistry staining images of p-AKT, p-JNK, and p-ERK in patient’s tumor tissues (magnification, ×200). **(F)** Total proteins were collected from the treated DH82 cells, and Western blot analyses for the expression of p-AKT, AKT, p-JNK, JNK, p-ERK, and ERK were performed. GAPDH was used as a loading control. Data were obtained from three independent experiments. ^***^ *p* < 0.001 vs. each control group.

To explore the possible underlying mechanism of imatinib plus thalidomide in this HS patient, experiments *in vitro* were performed in a canine HS cell line DH82. Results of cell counting kit-8 (CCK8) assays showed that the proliferation activity of DH82 was significantly inhibited by imatinib but not thalidomide (**Figures 3A, B**). Combined thalidomide and imatinib treatment did not improve the inhibitory effects of imatinib to DH82 (**Figure 3C**). We speculated that no synergistic effect existed between imatinib and thalidomide, but each of them might have its own specific antitumor activity.

As mentioned above, mutations of DUSP2 and GRIN2A are involved in the RET signaling pathway. RET signaling leads to the activation of the RAS/MAPK and the PI3K/AKT pathways and has key roles in cell growth, differentiation, and survival (8). Further KEGG pathway enrichment analysis of NGS results from patient’s tissue also revealed that PI3K/AKT and MAPK pathways were activated in this HS patient (**Figure 3D**). Immunohistochemistry staining on the patient’s tissue was performed to detect phosphorylated ERK (p-ERK) and phosphorylated JNK (p-JNK) of MAPK pathway and phosphorylated AKT (p-AKT) of PI3K/AKT pathway. Results showed that p-AKT and p-ERK were strongly positive, while p-JNK was almost negative (**Figure 3E**), indicating the patient actually harbored the activation of MAPK and PI3K/AKT pathways. Treatment of DH82 with imatinib demonstrated that p-ERK and p-AKT were substantially inhibited with imatinib while p-JNK was slightly elevated in a dose-dependent manner, which confirmed the inhibitory effects of imatinib on DH82 by targeting activation of MAPK and PI3K/AKT pathways (**Figure 3F**).

## Discussion

Consistent with previous reports, activating mutations of the RAS/MAPK and PI3K/AKT/mTOR pathways have been identified in a majority of HS cases and play a cooperative role in the pathogenesis of HS (9, 10). There are some published cases showing favorable response with targeting RAS/MAPK pathway by trametinib (MEK1/2 inhibitor) (5), crizotinib (MET and ALK inhibitor) (11), and vemurafenib (BRAF inhibitor) (12). Some scattered reports show imatinib is a promising agent that may exert a therapeutic benefit in HS (5, 7). Thalidomide also has a certain role in controlling tumors by regulating immune system (13). In the present case, imatinib plus thalidomide showed dramatic efficacy in HS. Our experiments *in vitro* revealed that it was imatinib, but not thalidomide, that inhibited the proliferation of HS and inhibited the MAPK and PI3K/AKT pathways.

The present case highlights the clinical importance of the multidisciplinary treatment. Chen et al. reported a case with localized disease which was successfully treated with a combination of CHOEP and radiotherapy (3). In this case, combined radiotherapy and targeted therapy (crizotinib) showed a remarkable local control and remission of symptoms due to the compression of the lesions in a short time. Continued 2-year imatinib plus thalidomide treatment provided the patient with a complete and durable remission. Radiotherapy combined with targeted therapy guided by NGS may be promising, and further perspective clinical trial is warranted for the localized HS.

## Data Availability Statement

The datasets presented in this article are not readily available due to ethical and privacy restrictions. Requests to access the datasets should be directed to the corresponding author.

## Ethics Statement

The studies involving human participants were reviewed and approved by the ethics committee of Union Hospital, HUST. The patient provided the written informed consent to participate in this study.

## Author Contributions

LZ conceived and designed the research. ZL and YX collected clinical data. ZL, YX, XL, QL, TL, FZ, GW, and LZ performed the research and/or analyzed data. ZL, YX, and LZ wrote the paper. All authors contributed to the article and approved the submitted version.

## Funding

This research was supported by grants from the National Natural Science Foundation of China (No. 81672940, 81300412) and the Clinical Research Physician Program of Tongji Medical College, Huazhong University of Science and Technology (No.5001530053).

## Conflict of Interest

The authors declare that the research was conducted in the absence of any commercial or financial relationships that could be construed as a potential conflict of interest.

## Publisher’s Note

All claims expressed in this article are solely those of the authors and do not necessarily represent those of their affiliated organizations, or those of the publisher, the editors and the reviewers. Any product that may be evaluated in this article, or claim that may be made by its manufacturer, is not guaranteed or endorsed by the publisher.
